# The skeletal proteome of the sea star *Patiria miniata* and evolution of biomineralization in echinoderms

**DOI:** 10.1186/s12862-017-0978-z

**Published:** 2017-06-05

**Authors:** Rachel L. Flores, Brian T. Livingston

**Affiliations:** Department of Biological Sciences, California State University, 1250 Bellflower Blvd, Long Beach, CA 90840 USA

**Keywords:** Skeleton, Proteome, Echinoderm, Deuterostome

## Abstract

**Background:**

Proteomic studies of skeletal proteins have revealed large, complex mixtures of proteins occluded within the mineral. Many skeletal proteomes contain rapidly evolving proteins with repetitive domains, further complicating our understanding. In echinoderms, proteomic analysis of the skeletal proteomes of mineralized tissues of the sea urchin *Strongylocentrotus purpuratus* prominently featured spicule matrix proteins with repetitive sequences linked to a C-type lectin domain. A comparative study of the brittle star *Ophiocoma wendtii* skeletal proteome revealed an order of magnitude fewer proteins containing C-type lectin domains. A number of other proteins conserved in the skeletons of the two groups were identified. Here we report the complete skeletal proteome of the sea star *Patiria miniata* and compare it to that of the other echinoderm groups.

**Results:**

We have identified eighty-five proteins in the *P. miniata* skeletal proteome. Forty-two percent of the proteins were determined to be homologous to proteins found in the *S. purpuratus* skeletal proteomes. An additional 34 % were from similar functional classes as proteins in the urchin proteomes. Thirteen percent of the *P. miniata* proteins had homologues in the *O. wendtii* skeletal proteome with an additional 29% showing similarity to brittle star skeletal proteins. The *P. miniata* skeletal proteome did not contain any proteins with C-lectin domains or with acidic repetitive regions similar to the sea urchin or brittle star spicule matrix proteins. MSP130 proteins were also not found. We did identify a number of proteins homologous between the three groups. Some of the highly conserved proteins found in echinoderm skeletons have also been identified in vertebrate skeletons.

**Conclusions:**

The presence of proteins conserved in the skeleton in three different echinoderm groups indicates these proteins are important in skeleton formation. That a number of these proteins are involved in skeleton formation in vertebrates suggests a common origin for some of the fundamental processes co-opted for skeleton formation in deuterostomes. The proteins we identify suggest transport of proteins and calcium via endosomes was co-opted to this function in a convergent fashion. Our data also indicate that modifications to the process of skeleton formation can occur through independent co-option of proteins following species divergence as well as through domain shuffling.

**Electronic supplementary material:**

The online version of this article (doi:10.1186/s12862-017-0978-z) contains supplementary material, which is available to authorized users.

## Background

Proteomic analysis of the matrices that mineral is deposited on during biomineralization has revealed complex mixtures of proteins. Among the more prominent proteins identified are groups of proteins that have evolved through gene duplication and concerted evolution of repetitive regions [1–6]. Although such proteins exist in disparate animal groups and share certain properties, they appear to have evolved independently [5, 6]. Also identified in skeletal proteomes have been proteins not previously thought to be involved in biomineralization or that have identified functions not previously associated with this process [7, 8]. In order to understand biomineralization we need to characterize what is necessary for it to occur and assign functional roles to the components that have been identified. Doing so may also shed light on how the process evolved. Comparative proteomics, as previously discussed by Degnan et al. [9], can provide information relevant to these questions.

Sea urchins have been a useful system to study skeleton formation during development. The cells that give rise to the skeleton appear early during cleavage and are easily studied both within the embryo and in culture. These cells produce an organic matrix upon which calcium carbonate is deposited to form the skeleton [10]. Proteomic studies on this matrix has been carried out for both adult and embryonic tissues and have identified a large number of proteins [11–13]. Among the most prevalent proteins found are the spicule matrix proteins. These are secreted proteins characterized by a C-type lectin domain and a series of acidic amino acid repeats enriched in proline and glycine [14]. Among other prevalent proteins are a membrane bound protein MSP-130 and carbonic anhydrase [11–13]. Studies of other sea urchins within the largest extant group, the euechinoids, shows these proteins to be conserved, although the exact sequence of the spicule matrix repeats varies [14]. The functional importance of individual spicule matrix proteins has been called into question [15], perhaps indicating functional redundancy within this group of proteins.

We have recently carried out proteomic analysis of the skeletal proteome of two brittle stars [7, 8]. We found several C-type lectin proteins in the skeletal proteomes, but these lacked the repetitive domains seen in the sea urchin spicule matrix proteins. The sequences of the proteins found in the two brittle stars were highly conserved. The brittle star and sea urchin proteins formed separate groups, and it was unclear whether they were derived from a common ancestral protein, or if the C-type lectins evolved a mineralization function separately in the two echinoderm groups. Absent from the brittle star skeletal proteomes was MSP-130. We also found a number of proteins not previously implicated in echinoderm skeleton formation that were in common between the two brittle stars and sea urchins. The relevance of these proteins to biomineralization is not clear, but they are conserved. Interestingly, many of these proteins have also been found in vertebrate bone proteomes [16] and the proteomes of matrix vesicle [17, 18], which are involved in deposition of vertebrate bone.

The observed differences between sea urchins and brittle stars led us to examine a third echinoderm group. In this study we describe the skeletal proteome of the sea star *Patiria miniata*. Sea stars diverged from sea urchins some 500 mya and are thought to form a clade with brittle stars [19]. The genome of *P. miniata* is available [20], allowing a comparison to an extensive list of gene models and computational predictions of peptide sequences. Surprisingly, we did not detect any C-type lectins in the *P. miniata* skeletal proteome, or MSP-130 like proteins. We did identify proteins that are conserved between all three echinoderm groups, as well as proteins unique to the sea star. Some of these proteins we identified have been determined to be present in vertebrate skeletal proteomes. We discuss the implications of our findings on the evolution of biomineralization in deuterostomes.

## Results and discussion

**Fig. 1:**
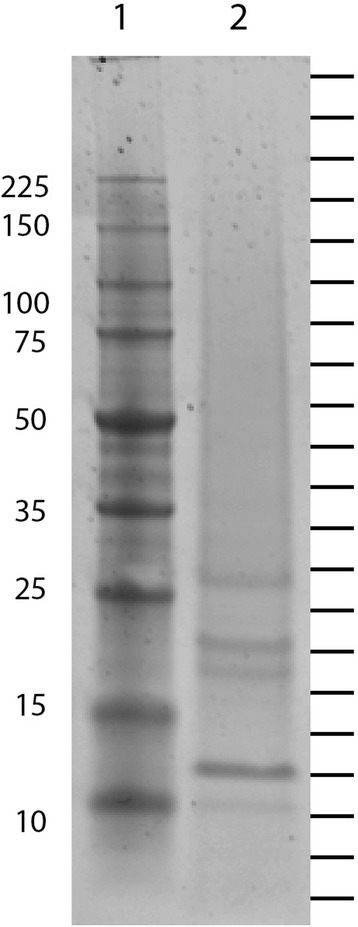
SDS-PAGE gel of *Patiria miniata* skeletal proteins. Lane 1; Molecular weight markers, Lane 2: *P. miniata* proteins. Molecular weights of marker bands are indicated on the left of the gel. The position of gel fractions separated prior to LC/MS/MS are indicated on the right of the gel

Skeletal elements from entire *Patiria miniata* adults were isolated collectively. Skeletal proteins isolated from clean skeletal preparations were separated by SDS-PAGE and fractionated into twenty equal slices (Fig. 1). Following tryptic digestion and LC-MS-MS analysis the peptide sequences were compared to the complete set of proteins computationally identified from the *Patiria miniata* genome sequence, which includes 29,697 annotated genes [20, 21]. A total of 8654 spectra yielded 517 unique peptides (Additional file 1). Proteins with at least two peptide matches and a minimum protein value indicating 95% identification certainty were accepted. After removal of peptides with internal stop codons or short reading frames these peptides matched 85 proteins in the *P. miniata* genome (Additional file 2, 20). All of these matched sequences in the NCBI database, although nine proteins match proteins of unknown function (Tables 1, 2 and 3). Of the nine uncharacterized proteins, three were homologous to proteins found in the *S. purpuratus* skeleton; the other four had homologues in the *S. purpuratus* genome. The number of proteins identified is similar to what was found in the brittle star *Ophiothrix spiculata* [8], but fewer than what has been identified in *S. purpuratus* skeletal proteomes. The test and spine proteomes of *S. purpuratus* contained 103 proteins combined. Over 400 proteins were found cumulatively in the *S. purpuratus* mineralized tissues, test, spine, tooth and larval spicules [11–13]. It should be noted that the number of different functional classes of proteins found in the skeletal proteomes do not differ significantly. In *S. purpuratus* there are more versions of each of the proteins from any particular functional class.

**Table 1:** *Patiria miniata* skeletal proteins with homologues in *Strongylocentrotus purpuratus* skeletal proteomes

		S. pupuratus protein	*S. purpuratus* Skeletal Proteome
	Fibrinogen C domains		
PMI_010282	fibrinogen C domain-containing 1-A-like	XP_783504.3	Spicule/Tooth
	Frem/Fras Proteins		
PMI_005627	extracellular matrix protein 3-like	XP_011665059.1	All
PMI_019827	extracellular matrix protein 3-like	XP_003724641.1	All
PMI_023895-RA	extracellular matrix protein 3-like	XP_011665059.1	All
	Cub domains		
PMI_001156	tolloid-like protein 2-like	XP_783971.3	Test/Spine
PMI_025317	Cub and sushi domain containing protein	NP_001001477.1	Test
	Alpha-2 macroglobulin-like		
PMI_010745	CD109 antigen-like	XP_011679264.1	All
	Ldlr-like		
PMI_002582	low-density lipoprotein receptor-related protein 5-like	XP_011680982.1	Spicule
PMI_001360	low-density lipoprotein receptor-related protein 5-like	XP_011680982.1	Spicule
PMI_016447	low-density lipoprotein receptor-related protein 5-like	XP_011678204.1	All
	Semaphorin		
PMI_019742	semaphorin-1A-like isoform 2	XP_11662560.1	All
	Matrix Metalloproteinases		
PMI_022451	MMP	XP_780356.3	All
	EGF domains		
PMI_000613	multiple epidermal growth factor-like domains protein 6-like	XP_001181407.3	Tooth
PMI_009988	contactin-associated protein-like 5-like	XP_781951.3	Tooth/Test
	Scavenger Receptor domains		
PMI_006935	Scavenger Rceptor/WSC/Sushi domains	XP_003729128.1	Test
PMI_007117	deleted in malignant brain tumors 1 protein-like	XP_011672690.1	Spicule
PMI_020133	scavenger receptor cysteine-rich domain superfamily protein-like	XP_011665404.1	All
PMI-005504	Scavenger receptor cysteine rich	XP_011665404.1	Spicule, tooth
	Miscellaneous Proteins		
PMI_001485	ATP synthase	NP_001116974.1	Spicule
PMI_002339	thrombospondin type-1 domain-containing protein 7A–like	XP_011661073.1	Test
PMI_013539	S. purp Cathepsin-like	XP_011676335.1	Spine
PMI_013990	uncharacterized protein LOC100889419	XP_003728419.1	Test/Spine
PMI_015421	carbonic anhydrase	XP_11681575.1	All
PMI_024890	Tetraspanin	NP_001118229.1	Spine
PMI_025657	calmodulin-1-like	XP_780862.3	All
PMI_027849	Sodium CA exchanger	XP_794875.3	Test
PMI_027881	Phospholipase A2	XP_003729200.1	All
PMI_006886	uncharacterized protein LOC578177 isoform 1	XP_011668665.1	Test/Spine
PMI_018767	uncharacterized protein LOC578177 isoform 3	XP_011668665.1	Test/Spine
PMI_022370	uncharacterized protein LOC578177 isoform 1	XP_011668665.1	Test/Spine
PMI_018059	MAM domain	XP_011660715.1	Spicule
PMI_006935	uncharacterized protein LOC100891625	XP_003729128.1	Test/Spine
	Cytoskeleton		
PMI_015982	actin related protein 1	NP_999634.1	Spine/Tooth
PMI_022236	alpha-2 collagen	NP_999675.1	Test/Spine, Spicule
PMI_002180	alpha-2 collagen	NP_999675.1	Test/Spine, Spicule
PMI_017378	alpha-2 collagen	NP_999675.1	Test/Spine, Spicule

**Table 2:** *Patiria miniata* skeletal proteins similar to proteins in *Strongylocentrotus purpuratus* skeletal proteomes

	Fibrinogen C domains	S. purpuratus protein
PMI_004711	fibrinogen C domain-containing 1-A-like	XP_011672460.1
PMI_010283	tenascin-R-like	XP_011677319.1
	Alpha-2 macroglobulin-like	
PMI_002096	pregnancy zone protein-like	XP_011669169.1
PMI_006509	pregnancy zone protein-like	XP_011675681.1
PMI_018900	Strumpellin	SPU_006076
PMI_002094	Alpha-2 macroglobulin-like	XP_011675682.1
	Ldlr-like	
PMI_004752	low-density lipoprotein receptor-related protein 5-like	XP_0116651661
PMI_027828	Cub/Ldlr domains	XP_001180573.3
	Semaphorin	
PMI_004864	semaphorin-6D	XP_011683607.1
	EGF domains	
PMI_001247	sushi, von Willebrand, EGF domain-containing protein 1-like	XP_003728106.1
PMI_004789	PREDICTED: stabilin-2-like	XP_011664162.1
PMI_024452	fibropellin-1 precursor	NP_001229629.1
	Kazal Protease Inhibitors	
PMI_025317	Sushi/Kazal domains	
	Scavenger Receptor domains	
PMI_000035	deleted in malignant brain tumors 1 protein-like	XP_011674594.1
PMI_003251	scavenger receptor cysteine-rich protein type 12 precursor	NP_999762.1
PMI_007118	scavenger receptor cysteine-rich protein type 12 precursor	NP_999762.1
PMI_008404	deleted in malignant brain tumors 1 protein-like	XP_011669254.1
PMI_012328	deleted in malignant brain tumors 1 protein-like	XP_011678662.1
PMI_016172	deleted in malignant brain tumors 1 protein-like	XP_011675091.1
PMI_017807	IG/Fugu/Scavenger Receptor Domains	XP_011669254.1
PMI_023266	deleted in malignant brain tumors 1 protein-like	XP_011679402.1
PMI_022661	scavenger receptor cysteine-rich protein type 12 precursor	NP_999762.1
	Matrix Metalloproteinases	
PMI_025070	matrix metalloproteinase 14 precursor	NP_001028823.1
PMI_017395	ADAMTS-like protein	XP_003726388.1
	Enzymes	
PMI_008848	aminopeptidase N isoform 2	XP_011665952.1
PMI_025324	Endothelin converting enzyme	XP_001191766.2
	MISC. Proteins	
PMI_014267	integrin beta G subunit precursor	NP_999732.1
PMI_008915	Vitellogenin	XP_003726749.1
PMI_010523	Fras-like	XP_011404569.1

**Table 3:** *Patiria miniata* skeletal proteins with no counterparts in *Strongylocentrotus purpuratus* skeletal proteomes

	VWC domains	S. purpuratus protein
PMI_019614	kielin/chordin-like protein-like	XP_792448.3
PMI_009987	kielin/chordin-like protein-like	XP_792448.3
PMI_018567	kielin/chordin-like protein-like	XP_792448.3
PMI_013233	kielin/chordin-like protein-like	XP_792448.3
PMI_003133	kielin/chordin-like protein-like	XP_792448.3
	Enzymes	
PMI_025303	lysozyme 3-like	XP_788343.1
PMI_012571	lysozyme 3-like	XP_788380.2
PMI_006134	alpha-amylase-like	XP_787209.3
PMI_008987	Peroxisomal Biogenesis	None
	MISC. Proteins	
PMI_025525	uncharacterized protein LOC100889940, partial	XP_011664341.1
PMI_006431	uncharacterized protein LOC590178	XP_011671697.1
PMI_026044	uncharacterized protein LOC 590178	XP_002610978.1
PMI_008152	uncharacterized protein LOC 100893135	XP_003723438.1
PMI_0019169	TGF beta receptor-like	XP_785846.2
PMI_017528	Complement C3 (alpha 2)	NP_9996086
PMI_003733	HHIP protein	XP_011682570.1
PMI_011218	double zinc ribbon ankyrin	XP_011682699.1
PMI_026044	WAP domains	XP_011682990.1
PMI_001641	Sushi/VWA/EGF domains	XP_011674476.1
PMI_021107	Mucin-like	XP_011667210.1

A comparison with the proteins found in the *S. purpuratus* skeletal proteomes identified thirty six proteins (42%) in *P. miniata* that have orthologues in *S. purpuratus* (Table 1) as defined by proteins found in the skeletal proteomes that are the top hits in reciprocal best blasts. In addition, we found twenty nine *P. miniata* skeletal proteins (34%) that were most similar to *S. purpuratus* proteins that are of the same functional class as proteins in the *S. purpuratus* skeletal proteomes, but the top protein identified by BLAST was not the one found in the *S. purpuratus* skeletal proteome (Table 2). We also identified twenty proteins (24%) that are unique to the *P. miniata* skeletal proteome (Table 3). Comparison to the two brittle star total adult skeletal proteomes that have been characterized [7, 8] identified twenty-one proteins (28%) of the *P. miniata* skeletal proteins were similar to those found in *Ophiocoma wendtii* and thirty-one (44%) with *Ophiothrix spiculata*.

Notably absent from the *P. miniata* skeletal proteome were C-type lectin proteins which are abundant in the *S. purpuratus* skeletal proteomes [11–13] and are present in both brittle star skeletal proteomes as well [7, 8]. MSP-130, another protein prevalent in the *S. purpuratus* skeletal proteomes was also absent, as it was in the brittle star skeletal proteomes. In order to confirm that we did not miss any C-type lectins, with or without the spicule matrix protein repeats, we used the *S. purpuratus* spicule matrix protein sequences as well as those encoding the brittle star *O. wendtii* skeletal C-type lectins to performed a BLAST search against the *P. miniata* genome sequences and predicted peptides [22]. We found that there are C-type lectins encoded in the genome, but nothing with repetitive domains resembling the sea urchin spicule matrix proteins. The C-type lectins identified in the *P. miniata* genome were most similar to the brittle star sequences. OwSM20 identified a group of proteins with an E-value high of e-22 and OwSM24 identified proteins with an E-value ranging up to e-20. Using the BLAST results, we examined proteins identified with single peptide hits and lowered the minimum protein value to 20%. We searched these proteins for the top 90 of the C-lectin proteins identified in the BLAST search. We identified two C-type lectin domain containing proteins in the *P. miniata* skeletal proteome. One, PMI_018577 had a single peptide hit and a 61% identification probability. It has two C-type lectin domains and very little similarity with spicule matrix proteins (e^−19^). The other, PMI_003522, had one peptide hit and a 41% identification probability. This protein also had additional Cub and FN3 domains not found in spicule matrix proteins. We also used *S. purpuratus* MSP-130 sequences to query the *P. miniata* genome and identified two proteins (PMI_017126 and PMI_019914) similar to MSP 130. We examined our LC/MS/MS data under relaxed stringency (one peptide match, <20% certainty) and could not find any matches to these two proteins. Computational analysis indicated trypsin digests would produce peptides that could be identified. MSP 130 is thought to be associated with the membrane and function in calcium transport. It is possible it is expressed in the skeleton forming cells in *P. miniata,* but is not incorporated into the skeleton in sea star adult skeletons. It is also possible that the gene annotations available missed the C-lectin and MSP 130 proteins in the skeletal proteome. 29,697 gene models were identified computationally [20]; however, the available genome is somewhat fragmented (N50 = 52 kb), which may overestimate the number of genes identified. Proteins similar to these are present in the *Patiria miniata* genome, but were not detected using LC/MS/MS analysis of the proteins we isolated from the skeleton. It is possible our methods failed to detect these proteins, however, at a minimum it suggests that C-type lectins and MSP 130 proteins are much reduced in the *P. miniata* skeletal proteome, if they are present at all.

**Fig. 2:**
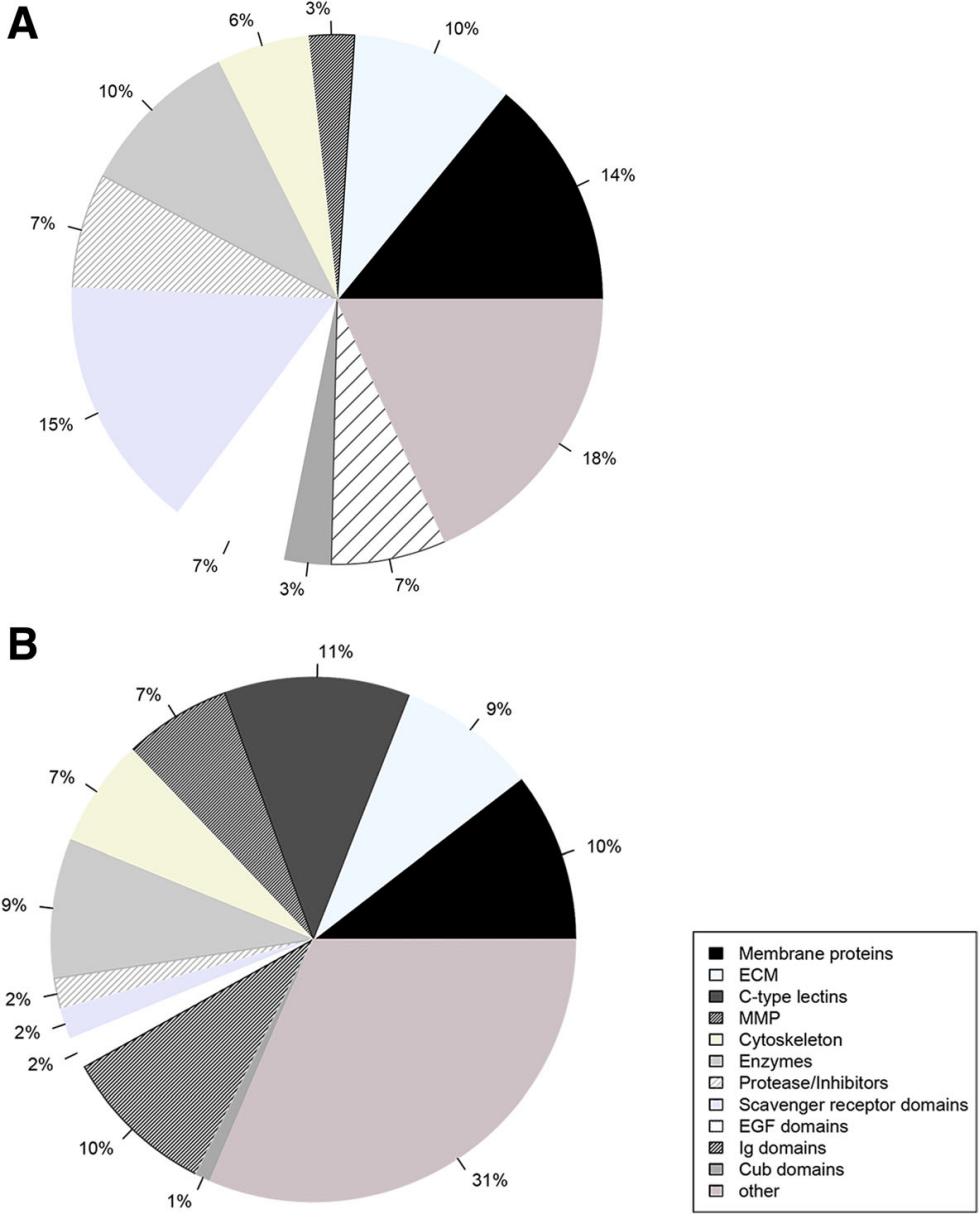
Functional classes of proteins identified in the skeletal proteomes of sea stars and sea urchins. **a** The functional classes of proteins in the *Patiria miniata* skeletal proteome. **b** The functional classes of proteins found in the *Strongylocentrotus purpuratus* test and spine skeletal proteome

The distribution of proteins into functional groups in members of sea stars and sea urchins are shown in Fig. 2. The types and distribution of proteins found in the skeleton are similar overall with some notable exceptions. Membrane, extracellular matrix, cytoskeletal proteins and proteins with enzymatic functions are present in similar proportions. The *S. purpuratus* skeletal proteome has more matrix metalloproteases, while the *P. miniata* skeletal proteome has more proteases and protease inhibitors, proteins with EGF domains, cub domain containing proteins and a large increase in scavenger receptor domain containing proteins. The greatest divergence is the presence of the large number of C-type lectin domain containing proteins in *S. purpuratus* that are missing in *P. miniata*, and the presence of Von Willebrand type C domain containing proteins in *P. miniata* that are missing in *S. purpuratus*.

*S. purpuratus* forms a skeleton in the embryo and during metamorphosis forms distinct test and spines. The proteomes of these skeletal elements in *S. purpuratus* have been studied separately. The types of proteins observed did not differ substantially between these tissues [11–13], although the spicule did contain some unique versions of protein families (e.g. SM30 and MMP’s) and there are similar differences between test and spine. In contrast, *P. miniata* does not form a skeleton in the embryo or larva. The skeleton formed at metamorphosis and in the adult has many smaller skeletal elements that are not as easily separated into classes. When comparing the proteins found in the *P. miniata* adult with the different proteomes of *S. purpuratus*, most of the homologous proteins in *P. miniata* were present in all of the *S. purpuratus* proteomes or in the adult skeleton (Table 1). There were a few exceptions to this. In some cases, multiple proteins from a functional group were found, and some of these were found in all of the *S. purpuratus* proteomes, while others were specific to the *S. purpuratus* spicule, for example Ldlr-like and scavenger receptors proteins. Two were uniquely found in the sea urchin spicule. One is the fibrinogen C-like -domain containing protein PMI_010282. In *S. purpuratus* this protein is only found in the larval skeleton and has been shown to be expressed at the growing ends of the spicule [23]. The other is the MAM domain containing protein PMI_018059 similar to meprin. This is an extracellular domain thought to have an adhesive function. It is possible that activation of the gene regulatory network leading to skeleton formation in embryos in *S. purpuratus* has allowed some degree of specialization of skeletal proteins in the embryo versus the adult. This specialization of proteins would not be expected in sea stars, which only make an adult skeleton. The presence of proteins similar to both sea urchin adult and spicule skeletons in adult sea stars supports that.

### Proteins conserved in echinoderm skeletal proteomes

#### *Scavenger Receptor domain containing* proteins

The protein with the highest spectral counts in the *P. miniata* skeletal proteome contained multiple scavenger receptor domains. The *S. purpuratus* protein with the highest similarity to this protein is not present in the skeletal proteome [11–13], however there are several proteins with scavenger receptor domains present in the *S. purpuratus* skeletal proteomes. Similar proteins were not detected in the brittle star skeletal proteomes [7, 8]. *P. miniata* has a large number of proteins with these domains in the skeletal proteome (Tables 1 and 2), some in combination with other domains such as IG domains (PMI_017807, Table 2). Scavenger receptor domains are cysteine rich and have been implicated in binding various ligands [24]. The most prevalent protein with multiple scavenger receptors in the *P.miniata* skeletal proteome predicted to be 415 kd in size and was found in the fraction of the gel (Fig. 1) consistent with being of large molecular weight.

#### Fibrinogen C-like proteins

**Fig. 3:**
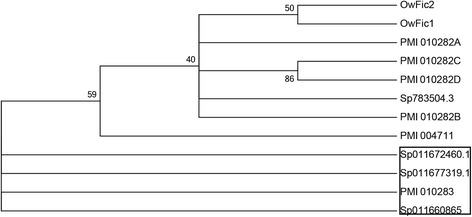
Phylogenetic relationship of echinoderm fibrinogen C domain containing proteins. A maximum likelihood tree was produced using proteins found in *Patiria miniata* = PMI, *Ophiocoma wendtii* = Ow and *Strongylocentrotus purpuratus* = Sp. PMI_010282 contains four domains which were separated into A, B, C and D for this analysis. All proteins used are found within the skeletal proteome except for the *S. pupuratus* proteins indicated by the box

All three echinoderm groups studied have proteins related to fibrinogen in their skeletal proteomes. In the brittle stars [7, 8] and *P. miniata*, these proteins are among the most abundant in the skeleton as inferred from total spectral counts. In *S. purpuratus* a similar protein was less abundant and was detected only in the spicule and tooth. The sea urchin fibrinogen C-like gene(frp) has been shown to be expressed in the larval skeleton [23]. The similarity to fibrinogen is found in the Fred domain that is found near the C-terminus of fibrinogen. These domains in vertebrate fibrinogen participate in lattice formation during blood clotting. The echinoderm proteins lack the collagen-like N-terminal domain of vertebrate fibrinogen C. The brittle star and sea urchin proteins contain a single Fred domain, while *P. miniata* has proteins with one and four Fred domains. The relationship between these proteins and the individual domains are shown in Fig. 3. The four FRED domains in the *P. miniata* protein PM 010282 were separated in silico and aligned with the other *P. miniata* Fic C protein found in the skeleton (PM 004711) and the *S. purpuratus* and *O. wendtii* skeletal fibrinogen C-like proteins, as well as some related *S. purpuatus* proteins not found in the skeleton. All but one of the Fibrinogen C proteins found in echinoderm skeletal proteomes clustered together, suggesting they are derived from an ancestral gene involved in skeleton formation. The larger, most prevalent *P. miniata* protein with four FRED domains is predicted to be 90 kd in size. It was detected in a gel fraction consistent with that, but was also found at lower molecular weights down to around 20 kd, which is consistent with a single domain and falls in fractions containing major bands on the gel (Fig. 1). This suggests the protein might be proteolytically processed.

#### Proteases and protease inhibitors

**Fig. 4:**
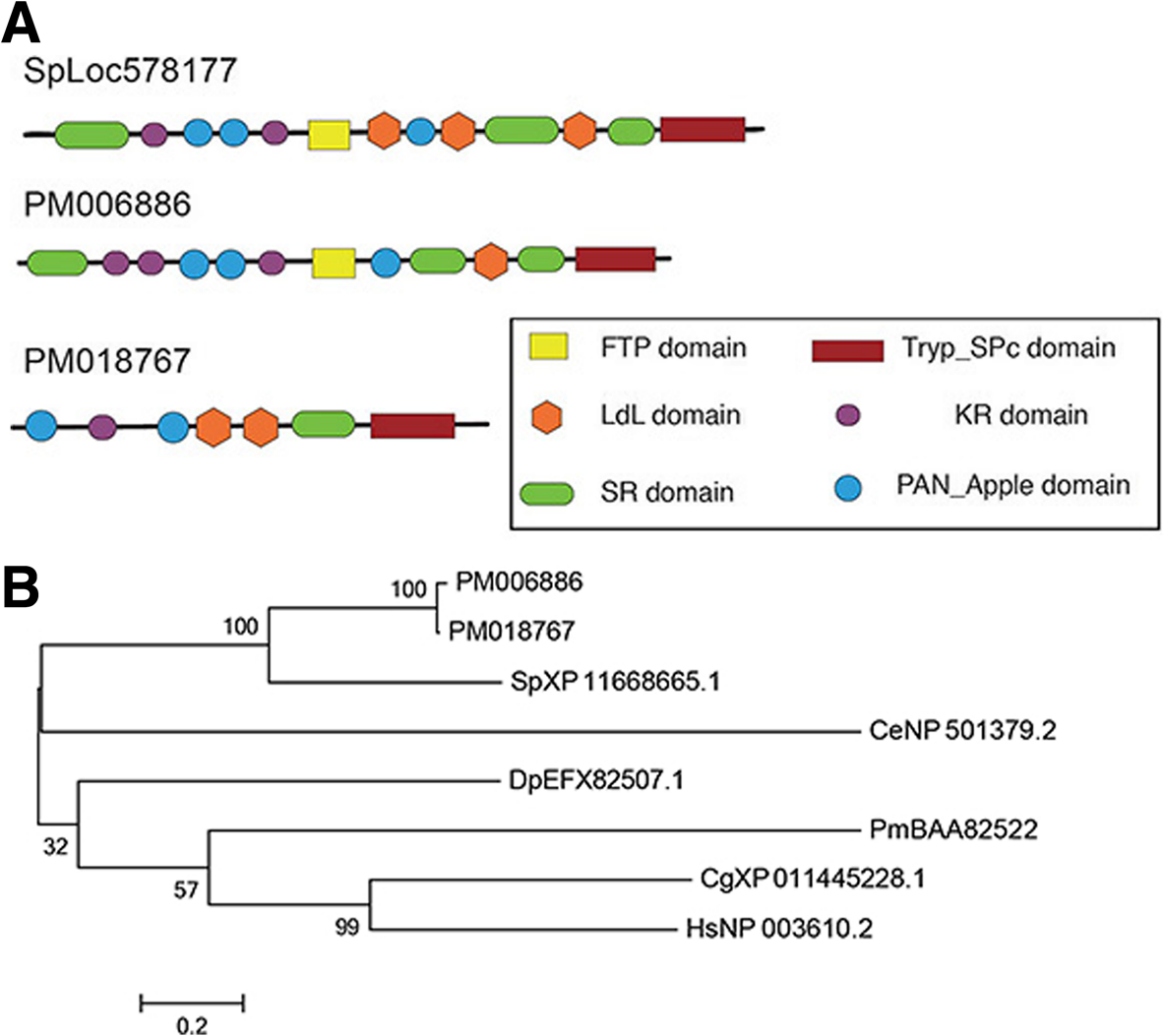
Domain structure and relationship to similar proteins of a novel protease conserved in echinoderm skeletal proteomes. **a** Domain organization of the *S. purpuratus* and *P. miniata* proteins. **b** A maximum likelihood tree showing related proteases in multiple species. PM = *Patiria miniata*, SP = *Strongylocentrous purpuratus*, Pm = *Polyandrocarpa misakiensis*, Cg = *Crassostrea gigasI, Hs =* Homo sapiens, Dp *= Daphnia pulex,* Cs = *Caenorhabditus elegans*

The *S. purpuratus* skeletal proteomes contain a large number of secreted proteases and protease inhibitors [11–13]. Prominent among these are matrix metalloproteinases, alpha-2-macroglobulins and agrin/kazal inhibitors. Several of these urchin matrix metalloproteinases have been shown to be specifically expressed in the skeleton forming cells during embryonic development [25]. Representatives of these protein groups are also found in the brittle star skeletal proteomes [7, 8]. Two matrix metalloproteinases were found in the *P. miniata* skeletal proteome as well as four alpha-2-macroglobulin-like proteins (Tables 1 and 2). The most prevalent of these is homologous to MMP-14A and was found in the gel fraction where a major band of 28 kd was seen on the gel, consistent with the predicted molecular weight. A single protein with kazal inhibitor domains was detected. We also identified a novel protease that is present in all of the echinoderm skeletal proteomes examined. Our initial analysis using predicted peptides identified three *P. miniata* peptides similar to *S. purpuratus* LOC578177 (Table 1). A portion of this protein was identified in the *S. purpuratus* skeletal proteome using the original gene model that predicted it was a separate protein, but the current gene model has corrected that. A model of the domain structure is shown in Fig. 4. This figure also shows the relationship of this protein to the proteins it most closely resembles in Genbank databases. The only member of this group that has been characterized is human neurotrypsin, a secreted protease that cleaves proteoglycans and releases a signaling molecule [26]. Proteoglycans play an important role in formation of sea urchin skeletons [27], suggesting a potential role for this conserved protease.

#### Frem/Fras-like proteins

**Fig. 5:**
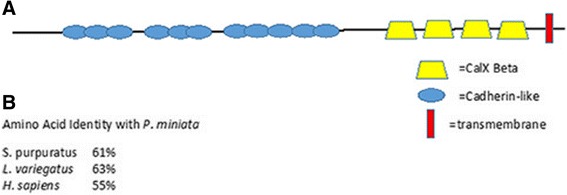
Conserved structure of Frem/Fras proteins found in echinoderm skeletal proteomes. **a** Domain structure of the *Patiria miniata* Fras homologue. **b** The amino acid identity of echinoderm and human Fras proteins compared to the *P. miniata* protein

*P. miniata* has three gene models that were identified as encoding peptides present in the skeletal proteome (Table 1). These actually encode a single protein that is homologous to a protein detected in all of the *S. purpuratus* skeletal proteomes [11–13]. The *S. purpuratus* gene encoding this protein was originally annotated as three separate genes but is now corrected in the NCBI database. This protein was originally reported in separate studies as a component of the embryonic extracellular matrix in the sea urchin *Lytechinus variegatus* and was called ECM3 [28, 29]. The protein is expressed by ectodermal cells in the sea urchin embryo and forms an extracellular complex with the highest concentration in the vegetal pole. The mesenchyme cells that form the skeleton interact with this protein [30]. These cells form an elongated syncytium, whose membrane surround a cylindrical, extracellular privileged space. The syncytial mesenchyme may engulf the ECM3 molecule as it forms the secluded space where the skeleton is formed. While ECM3 likely plays a role in skeletal patterning, its presence in the mineralized skeleton could be due to being trapped where mineralization occurs. In vertebrates the homologous protein (Fras or Frem) is found in the basal lamina and interacts with epithelial sheets [30]. Defects in the Fras/Frem protein lead to a condition called Fraser syndrome where epithelial sheets form blebs instead of interacting smoothly with the basal lamina. This protein is highly conserved, with over 60% of the amino acids conserved across echinoderms and hemichordates and over 50% conserved with humans (Fig. 5). The distinguishing features of the protein are numerous chondroitin sulfate proteoglycan repeats and five Calx-beta domains that have been implicated in sodium/calcium exchange. These results suggest this protein plays a role in ECM-membrane interaction during skeleton formation in echinoderms.

#### Ldlr-like (Lrp) proteins

**Fig. 6:**
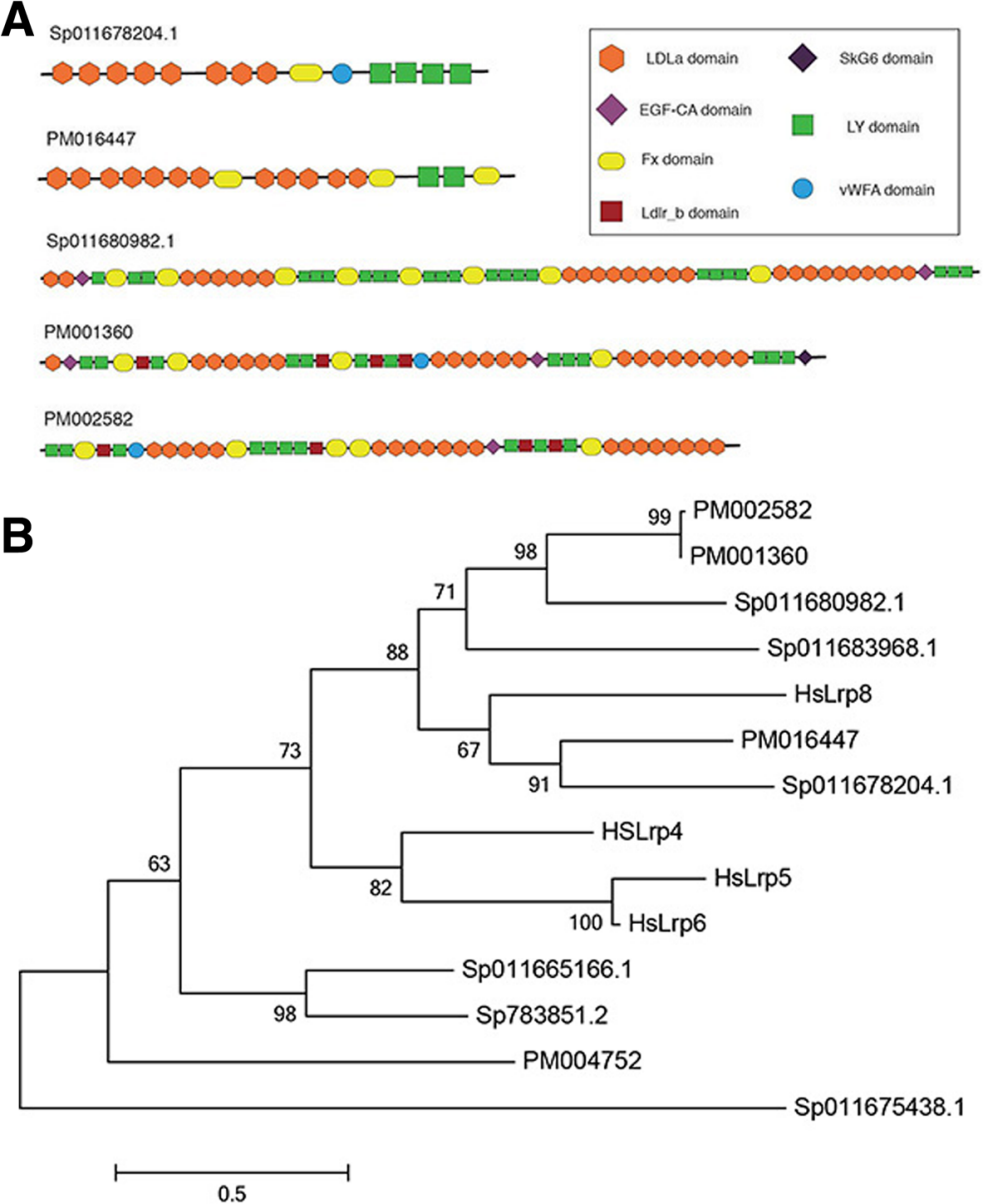
*Patiria miniata* (PM) Ldl-receptor-like proteins (Lrp). **a** Domain structure of the most prevalent Lrp-like proteins in *Patiria miniata* (PM) and *Strongylocentrotus purpuratus* (Sp). **b** A maximum likelihood tree of Lrp-like proteins found in skeletal proteomes compared to Human LRP proteins involved in bone formation. Hs = *Homo sapiens*

Proteins similar to low density lipoprotein-like receptors (Lrp) are found in the skeletal proteome of *P. miniata*, *S. purpuratus* [11–13] and both brittle stars [7, 8]. Similar proteins are found in vertebrate bone proteomes [16–18] and proteins with Lrp domains have now been identified in coral mineral proteomes [31]. Genes encoding Lrp proteins have been implicated in human osteoporosis through genome-wide association studies [32, 33]. The *P. miniata* skeletal proteome has four Lrp proteins, while the *S. purpuratus* skeletal proteomes have six, four in the test and spine [11–13]. Figure 6 shows the domain structure of the *P. miniata* proteins as well as the two most prevalent *S. purpuratus* proteins. The proteins have similar domains, but the number and order of the domains varies, suggesting duplication and rearrangement within the proteins. The phylogenetic relationship between the proteins is also shown in Fig. 6. The *S. purpuratus* Lrp protein (Sp011678204) that is present in all of the skeletal proteomes and is the most prevalent in test and spine [11] groups with the *P. miniata* PM016447 protein and human Lrp 8. The other prevalent *P. miniata* Lrp proteins group together with two *S.* pupuratus proteins found only in the larval spicule [13]. These proteins are also more similar to the human Lrp8 than to other human Lrp proteins involved in bone formation [34]. The Lrp proteins in vertebrates have been shown to be wnt co-receptors [35], but we have not found wnts or wnt receptor proteins in the echinoderm skeletal proteomes. This suggests they also may play separate roles during mineralization.

#### Other proteins

A variety of other proteins associated with mineralization in echinoderms, vertebrates or other animals were detected in the *P. miniata* skeletal proteome. These include carbonic anhydrase, calmodulin, and a protein orthologous to the sea urchin p58 protein which has been shown to play a role in skeleton formation [36]. The *P. miniata* skeletal proteome also contained a number of proteins that would not be expected to play a direct role in mineralization. These could be contaminants, although the stripping of organic material from the skeleton during isolation is extremely rigorous. It is also possible that these proteins are occluded within the mineral, but were trapped there because of involvement in cellular process involved with cell signaling, movement of materials into the extracellular space, membrane fusion, or proteolytic cleavage of ECM molecules, or are trapped in the organic matrix where mineralization occurs (e.g. ECM 3). When comparing different echinoderm skeletal proteomes, the presence of such proteins is not random; the same proteins are found in different classes of echinoderms. This includes cathepsin-like protein, alpha-2 macroglobulin, thrombospondin domain containing protein, actin and collagen (Table 1). Some proteins found in the echinoderm skeletal proteomes are not found occluded in vertebrate bone, but are associated with the process of mineralization, such as semaphorin, tetraspanin, a MAM domain containing membrane anchored protein related to meprin, a phospholipase and matrix metalloproteinases. In addition, there are a number of proteins that do not have exact homologues between the echinoderms and other metazoans examined, but that contain protein domains in various combinations that are prevalent in proteins found in all mineralized tissues. These include Cub, Egf, sushi, Ig, scavenger receptor and von willebrand factor type C (VWC) domains (Tables 1, 2 and 3). The *P. miniata* skeletal proteome has an expanded number of proteins with VWC and scavenger receptor domains.

## Conclusions

Matrix proteins have been studied in a variety of systems [1–9]. Many of the prevalent proteins that have been identified have similar characteristics but have evolved independently. They tend to be elongated, somewhat unstructured or flexible, glycosylated, acidic proteins. Often they contain repetitive, rapidly evolving domains which can vary in amino acid sequence even among related groups. The sea urchin spicule matrix proteins fit this description, and there are many in the urchin skeletal proteome [11–13]. These spicule matrix proteins also have a C-type lectin domain that binds carbohydrates. The large number of spicule matrix proteins with extensive acidic repeat domains in the skeleton seems to be unique to sea urchins among echinoderms. Brittle stars have C-type lectin proteins in their skeleton, but these lack the repetitive domains [7, 8]. The data we present here suggest sea stars do not have similar proteins in their skeletons. It seems that a meshwork of proteins and carbohydrates with appropriate charge is necessary to provide an environment conducive to nucleation and/or propagation of calcium crystals. The exact sequence of the proteins seems less important than the overall structure, such that the proper conditions can be reached with different combinations. There is evidence the sea urchin spicule matrix proteins form such a lattice [37]. The proteins in sea urchins [6, 11–14] molluscs [4, 5], and vertebrates [2, 3] that acquire repetitive domains seem well suited to biomineralization, such that they have been co-opted to this function numerous times and are highly prevalent in the skeleton. The appearance of the spicule matrix proteins in sea urchins likely supplanted proteins or reinforced proteins that formed an extracellular surface where mineral is deposited in ancestral echinoderms. A candidate for an ancestral protein that could also play this role is the fibrinogen C–like protein. This protein is one of the most abundant in the brittle star [7, 8] and sea star (ibid) skeletal proteome, but is much reduced in the sea urchin [11–13]. Similarly, the *P. miniata* proteome, which lacks C-type lectins, has a large number of proteins with VWC domains which are not found in the other echinoderm proteomes. These are extracellular proteins that could also play a similar role in forming a surface for mineral deposition.

While it is not unexpected to see proteins such as carbonic anhydrase or calmodulin conserved in skeletons among animals across a large phylogenetic distance, the conservation of a number of proteins seemingly unrelated to biomineralization is surprising. However, a number of these conserved proteins have been found in vertebrate matrix vesicles and are associated with formation and function of these exosomes in mineral deposition [17, 18, 38]. In vertebrates these matrix vesicles are released from the osteoblasts into the extracellular space where they contact collagen. Formation of the hydroxyapatite mineral is thought to begin within these vesicles [39]. As the crystals grow the vesicles burst and the mineral formation continues in the extracellular space. This can explain the presence of proteins occluded in bone that are also found in the matrix vesicles. Sea urchin embryonic micromere descendants have been shown to be full of multivesicular bodies as they form the skeleton [40] which resemble the formation of matrix vesicles. This, along with our findings of multiple proteins associated with vertebrate matrix vesicles in echinoderm skeletal proteomes, suggests that the role of exosomes in mineral deposition is conserved among deuterostomes.

An interesting feature of the mineral proteomes is the number of conserved protein domains that are found in different numbers, combination and arrangements. Apparently non-homologous proteins containing cub, sushi, Egf, scavenger receptor, Ig and von Willebrand domains in varying combinations are found in vertebrates [16–18], echinoderms [7, 8, 11–13], corals [32] and molluscs [4, 40]. This supports the idea put forth by Kocot et al. [41] that rapid evolution of these proteins involves extensive domain shuffling and duplication.

## Methods

### Protein purification

Skeleton was isolated as described in Seaver and Livingston and Flores et al. [7, 8]. Whole animals were cut into small pieces and immersed in 6% sodium hypochlorite. Skeletal elements were allowed to settle. The sodium hypochlorite was changed three times and the skeletal elements were then washed three times with sterile water. Removal of material adhered to the skeleton was monitored with microscopy. The skeleton was then powdered with a mortar and pestle. The powder was added to 4% guanidine isothiocyanate containing beta-mercaptoethanol and buffered with sodium citrate. Following settling of the mineral, the solution was replaced twice followed by three washes with sterile water. Purified skeletal elements were de-mineralized with acid and dialyzed as described previously [7, 8]. The soluble fraction was concentrated using Amicon Ultra-15 centrifugal filter units, followed by Amicon Ultra-0.5 centrifugal filter units (3000 NMWL) (Millipore, Bellerica, MA). Samples were dissolved in NuPAGE LDS sample buffer under reducing conditions (Invitrogen, Carlsbad, CA) and the proteins separated by SDS-PAGE using NuPAGE 4–12% Bis-Tris Polyacrylamide gels 1.0 mm*10 well in the MES buffer system (Invitrogen, Carlsbad, CA). Running times, protocols, and reagents were used as described by the manufacturer (Invitrogen, Carlsbad, CA). Gels were stained with SYPRO RUBY Protein Gel Stain.

### Sample preparation

Protein quantitation was performed by Qubit Fluorometry (Invitrogen, Carlsbad, CA). 20 μg of material was separated on a 4–12% Bis Tris NuPage gel (Invitrogen, Carlsbad, CA) in the MOPS buffer system. The gel lane was excised into twenty equally sized segments. Using a robot (ProGest, DigiLab), gel pieces were reduced with 8 mM dithiothreitol at 60 °C followed by alkylation with 10 mM iodoacetamide at RT. Samples were then digested with sequencing grade trypsin (Promega, Madison, WI) at 37 °C for 4 h and quenched with formic acid. The supernatant was analyzed directly without further processing.

### Mass spectrometry

For each sample (*n* = 24; from 3 excised gel slices, 1 unfractioned liquid sample, and 20 fractioned gel slices), LC-MS/MS was performed identically. The digested sample was analyzed by nano LC/MS/MS with a Waters NanoAcquity HPLC system interfaced to a ThermoFisher LTQ Orbitrap Velos. Peptides were loaded on a trapping column and eluted over a 75 μm analytical column at 350 nL/min; both columns were packed with Jupiter Proteo resin (Phenomenex). The mass spectrometer was operated in data-dependent mode, with MS performed in the Orbitrap at 60,000 FWHM resolution and MS/MS performed in the LTQ. The fifteen most abundant ions from LC of each gel slice were selected for MS/MS in each round run in continuous mode.

### Data processing

Data were searched using a local copy of Mascot against the predicted peptides from the *P. miniata* genome in Echinobase [20]. Trypsin was input as the digestive enzyme, mass values were monoisotopic, the peptide mass tolerance was 10 ppm, the fragment mass tolerance was 0.8 Da, and the maximum number of missed cleavages was 2. Carbamidomethylation (C) was set as a fixed modification and variable modifications included oxidation (M), acetyl (N-term), pyro-glu (N-term Q), and deamidation (N,Q). Mascot DAT files were parsed into the Scaffold software for validation, filtering and to create a non-redundant list per sample. The data were filtered using a minimum protein value of 95%, a minimum peptide value of 50% (Prophet scores) and requiring at least two unique peptide per protein.

### Phylogenetic analysis

Protein sequences were aligned in Clustal X and imported into Mega for refinement. Trees were constructed with maximum likelihood methods with 1000 bootstraps.

## Additional files

Additional file 1: Peptides identified by LC/MS/MS analysis. All MS/MS samples were analyzed using Mascot (Matrix Science, London, UK; version 2.4.0). Mascot was set up to search the P_Miniata_Custom_20130506 database [20] assuming the digestion enzyme stricttrypsin. (TXT 718 kb)

Additional file 2: Proteins identified from MS/MS analysis. Scaffold (version Scaffold_4.6.1, Proteome Software Inc., Portland, OR) was used to validate MS/MS based peptide and protein identifications. Peptide identifications were accepted if they could be established at greater than 50.0% probability by the Peptide Prophet algorithm with Scaffold delta-mass correction. Protein identifications were accepted if they could be established at greater than 90.0% probability and contained at least 2 identified peptides. Protein probabilities were assigned by the Protein Prophet algorithm. Proteins that contained similar peptides and could not be differentiated based on MS/MS analysis alone were grouped to satisfy the principles of parsimony. (TXT 80 kb)

## Abbreviations

*O. wendtii*: *Ophiocoma wendtii*
*P. miniata*: *Patiria miniata*
PAGE: Polyacrylamide Gel Electrophoresis
*S. pupuratus*: *Strongylocentrotus purpuratus*
